# Using the Chat Generative Pre-trained Transformer in academic writing in health: a scoping review

**DOI:** 10.1590/1518-8345.7133.4194

**Published:** 2024-06-14

**Authors:** Isabelle Cristinne Pinto Costa, Murilo César do Nascimento, Patrícia Treviso, Lucélia Terra Chini, Bartira de Aguiar Roza, Sayonara De Fátima Faria Barbosa, Karina Dal Sasso Mendes

**Affiliations:** 1 Universidade Federal de Alfenas, Escola de Enfermagem, Alfenas, MG, Brazil.; 2 Universidade do Vale do Rio dos Sinos, Escola de Saúde, São Leopoldo, RS, Brazil.; 3 Universidade Federal de São Paulo, Escola Paulista de Enfermagem, São Paulo, SP, Brazil.; 4 Universidade Federal de Santa Catarina, Departamento de Enfermagem, Florianópolis, SC, Brazil.; 5 Universidade de São Paulo, Escola de Enfermagem de Ribeirão Preto, PAHO/WHO Collaborating Centre for Nursing Research Development, Ribeirão Preto, SP, Brazil.

**Keywords:** Nursing, Artificial Intelligence, Scientific and Technical Publications, Writing, Research, Health Sciences, Enfermería, Inteligencia Artificial, Publicaciones Científicas y Técnicas, Escritura, Investigación, Ciencias de la Salud, Enfermagem, Inteligência Artificial, Publicações Científicas e Técnicas, Redação, Pesquisa, Ciências da Saúde

## Abstract

**Objective:**

to map the scientific literature regarding the use of the Chat Generative Pre-trained Transformer, ChatGPT, in academic writing in health.

**Method:**

this was a scoping review, following the JBI methodology. Conventional databases and gray literature were included. The selection of studies was applied after removing duplicates and individual and paired evaluation. Data were extracted based on an elaborate script, and presented in a descriptive, tabular and graphical format.

**Results:**

the analysis of the 49 selected articles revealed that ChatGPT is a versatile tool, contributing to scientific production, description of medical procedures and preparation of summaries aligned with the standards of scientific journals. Its application has been shown to improve the clarity of writing and benefits areas such as innovation and automation. Risks were also observed, such as the possibility of lack of originality and ethical issues. Future perspectives highlight the need for adequate regulation, agile adaptation and the search for an ethical balance in incorporating ChatGPT into academic writing.

**Conclusion:**

ChatGPT presents transformative potential in academic writing in health. However, its adoption requires rigorous human supervision, solid regulation, and transparent guidelines to ensure its responsible and beneficial use by the scientific community.

## Abstract (es)

**Objetivo:**

mapear la literatura científica sobre el uso del *Chat Generative Pre-trained Transformer* , ChatGPT, en la escritura académica en salud.

**Método:**

se trató de una revisión de alcance, siguiendo la metodología del JBI. Se incluyeron bases de datos convencionales y literatura gris. La selección de los estudios se realizó previa eliminación de duplicados y evaluación individual y en pares. Los datos se extrajeron basándose en un guión elaborado y se presentaron en un formato descriptivo, tabular y gráfico.

**Resultados:**

el análisis de los 49 artículos seleccionados reveló que ChatGPT es una herramienta versátil, que contribuye a la producción científica, descripción de procedimientos médicos y elaboración de resúmenes alineados con los estándares de las revistas científicas. Se ha demostrado que su aplicación mejora la claridad de la redacción y beneficia áreas como la innovación y la automatización. También se observaron riesgos, como la posibilidad de falta de originalidad y cuestiones éticas. Las perspectivas futuras resaltan la necesidad de una regulación adecuada, adaptación ágil y búsqueda de un equilibrio ético en la incorporación del ChatGPT a la escritura académica.

**Conclusión:**

ChatGPT presenta un potencial transformador en la escritura académica en el área de la salud. Sin embargo, su adopción requiere una supervisión humana rigurosa, una regulación sólida y directrices transparentes para garantizar su uso responsable y beneficioso por parte de la comunidad científica.

## Abstract (pt)

**Objetivo:**

mapear a literatura científica referente ao uso do *Chat Generative Pre-trained Transformer* , ChatGPT, na escrita acadêmica em saúde.

**Método:**

tratou-se de uma revisão de escopo, seguindo o método do JBI. Foram incluídas bases de dados convencionais e literatura cinzenta. A seleção dos estudos foi realizada após a remoção de duplicatas e avaliação individual e em pares. Os dados foram extraídos com base em um roteiro elaborado, e apresentados de forma descritiva, tabular e gráfica.

**Resultados:**

a análise dos 49 artigos selecionados mostrou que o ChatGPT é uma ferramenta versátil, que contribui para a produção científica, descrição de procedimentos médicos e elaboração de resumos alinhados aos padrões das revistas científicas. Sua aplicação melhorou a clareza da redação e beneficia áreas como inovação e automação. Também foram observados riscos, como a possibilidade de falta de originalidade e questões éticas. Perspectivas futuras destacam a necessidade de regulamentação adequada, adaptação ágil e busca por um equilíbrio ético na incorporação do ChatGPT na escrita acadêmica.

**Conclusão:**

o ChatGPT apresenta um potencial transformador na escrita acadêmica na área da saúde. Contudo, sua adoção requer supervisão humana rigorosa, regulamentação sólida e diretrizes transparentes para garantir seu uso responsável e benéfico pela comunidade científica.

## Introduction

 Contemporary digital health is influenced by technological advances, such as the Chat Generative Pre-trained Transformer (ChatGPT), within the scope of Artificial Intelligence (AI), which demonstrates potential for improving academic writing ^(^ 1 ^)^ . The definition of AI encompasses a multidisciplinary approach to creating machines capable of performing complex tasks, including natural language processing ^(^ 2 ^-^ 3 ^)^ . 

 ChatGPT is a language model, developed by the company OpenAI and launched on the market in November 2022. OpenAI is an AI research laboratory based in the United States, comprised of two institutions: a non-profit entity (OpenAI Incorporated) and a for-profit entity (OpenAI Limited Partnership). It is worth noting that there are two ways to access the ChatGPT artificial intelligence platform: free, with limited access tools and longer time to update the information that supplies the platform - the last one occurred in 2022; and paid access, without limitations (ChatGPT Plus), updated in 2023. ChatGPT, an evolution of the GPT-3 model, is specifically trained to generate responses in human language, and is applied in several areas, such as chatbots and automated writing ^(^ 2 ^-^ 4 ^)^ . 

 Chatbots are activated by a simple language instruction, also known as a “prompt”, provided by the user, and generate responses based on statistical and probabilistic language models ^(^ 5 ^)^ . They are widely adopted due to their ability to provide detailed answers, however, there are concerns regarding their ability to produce accurate scientific texts ^(^ 6 ^)^ . Studies indicate that ChatGPT can be a valuable auxiliary tool in academic writing, but human supervision is crucial to ensure its precision ^(^ 7 ^)^ . 

 Many scientific journals still do not recognize ChatGPT as an author of articles, thus highlighting the need for ethical guidelines and regulations for its responsible use ^(^ 8 ^-^ 10 ^)^ . Despite the concerns raised, a proper implementation of ChatGPT and other language models can accelerate innovation in healthcare and promote diversity in research, by eliminating language barriers ^(^ 7 ^,^ 11 ^)^ . 

Given the legitimate concerns presented regarding the potential inappropriate use of ChatGPT, it is of utmost importance to establish appropriate guidelines and regulations to ensure the safe and responsible utilization of artificial intelligence capabilities. This becomes essential to limit possible future complications and mitigate potential risks and negative results. As ChatGPT is increasingly adopted in the scientific community, particularly in healthcare, there is a pressing need to better understand its specific applications and contributions.

The selection of the approach to conduct the present study is justified by the aim of identifying the available evidence in a specific domain, which in this case refers to the use of ChatGPT in academic writing. Exploring the scientific literature in this area can reveal significant patterns, gaps, and ethical and legal implications, as well as possibilities and contributions of artificial intelligence technology, such as language models. Therefore, the present study aims to map the scientific literature regarding the use of ChatGPT in academic writing in health, with the aim of identifying trends, gaps and contributions to scientific knowledge. This will allow for a more in-depth understanding of the applications of this technology in contemporary times, as well as the associated ethical implications.

## Method

 This was a scoping review conducted according to the JBI methodology ^(^ 12 ^)^ . The research protocol was registered in the Open Science Framework under registration DOI 10.17605/OSF.IO/MDKHG ^(^ 13 ^)^ , and the PRISMA-ScR (Preferred Reporting Items for Systematic Reviews and Meta-Analyses for Scoping Reviews) extension was used to report the results of the scoping analysis ^(^ 14 ^)^ . 

### Eligibility criteria

Taking into account the acronym “Population, Concept and Context (PCC)” to formulate the research question, the following question was obtained: What is the current panorama of scientific literature in the health area that addresses the use of ChatGPT in academic writing, including its trends, gaps and contributions to current scientific knowledge?

Publications that focus on the use of ChatGPT in the production of academic writing in the health area were considered, excluding those studies that used writing tools or technologies other than ChatGPT. Regarding sources, the review encompassed experimental and quasi-experimental studies, analytical and descriptive observational studies, qualitative approaches, systematic reviews and meta-analyses. In addition, book chapters, conference summaries, theses, dissertations and other sources of gray literature relevant to the topic were considered, such as journals and websites specialized in the health area, in order to encompass a comprehensive range of perspectives and evidence.

### Information sources and literature search

 The search strategy sought to locate published and unpublished studies, including gray literature, in three stages. The first step involved an initial search in PubMed and the Cumulative Index to Nursing and Allied Health Literature (CINAHL) to find relevant articles on the topic. This included identifying keywords in titles and abstracts, as well as indexed terms (MeSH/CINAHL Headings), to develop a comprehensive search strategy ^(^ 15 ^)^ . 

The review team decided to focus only on terms related to ChatGPT in their search strategy, given the newness of the topic. The inclusion of all elements of the PCC strategy did not help to find relevant records about scientific writing, due to the current stage of publications on ChatGPT. Using more specific terms ensured a sensitive and comprehensive search, without losing the focus of the review. Therefore, the choice to use only the concept terms in the search strategy was appropriate for the objectives of this study.

The final search pilot was conducted in two databases, namely PubMed and Latin American and Caribbean Health Sciences Literature (LILACS). After identifying the relevance of the implemented strategy, as well as the possible recognition of new terms related to the ChatGPT concept, the protocol was registered. As an example, the terms used in the search strategy implemented in international databases are highlighted: ((“ChatGPT” OR “Chat GPT” OR “Generative Pre-trained Transformer” OR “Generative Pretrained Transformer” OR “GPT language model” OR “Transformer-based language models”) AND (“academic writing” OR “academic publications” OR “scientific writing” OR “scientific publications” OR “scholarly writing” OR “scholarly publications” OR “Writing for Publication” OR “text production” OR “computer-assisted writing” OR “virtual writing assistance” OR “virtual writing assistant” OR “writing automation” OR “natural language processing”)).

After this step, the definitive search was carried out on May 12, 2023, in the following databases: LILACS, PubMed, CINAHL, Embase, Scopus, Cochrane Database of Systematic Reviews and Web of Science Core Collection. Gray literature identification was searched on Google Scholar. Studies published in any language were included.

### Selection of evidence sources

 After searching the information sources, the identified citations were imported into the EndNote 20 software (Clarivate Analytics, PA, EUA), where duplicates were removed ^(^ 16 ^)^ . Then, the articles were exported to the Rayyan application (Rayyan Systems Inc., Cambridge, MA, USA), used to select the studies ^(^ 17 ^)^ . 

When selecting studies, two independent evaluators analyzed the titles and abstracts, following predefined criteria for inclusion. Subsequently, the full texts of potentially relevant studies underwent a thorough analysis by the same evaluators, maintaining the same inclusion criteria. All reasons for exclusion of studies that did not meet the criteria were documented in detail. Any disagreement between evaluators during the selection process was resolved through discussion or with the intervention of a third evaluator.

### Data extraction

 Two independent reviewers used a script in Microsoft Excel to extract data from the studies selected in the scoping review. The extracted data included identifying information such as authors, title, year, language, journal, institution and country. Objectives, methods and main results were also collected, when applicable, following the PCC structure. This step evaluated contributions from the literature, and identified limitations, gaps, emerging trends and practical implications of using ChatGPT in academic writing ^(^ 18 ^)^ . The data extraction form, as well as the guidance form for data extraction, detailing each item to be extracted, is presented in the Supplementary Material (available at: https://doi.org/10.48331/scielodata.BMQMKD ). 

### Data analysis and presentation

 When conducting data analysis, the basic qualitative content analysis method was used, involving a combined approach of inductive analysis, followed by deductive analysis. This method is widely recognized and used in qualitative research, as well as in scoping reviews ^(^ 15 ^,^ 19 ^)^ . 

 It began with an open coding process, in which researchers, in an impartial manner, identified pertinent concepts, themes and characteristics in the raw data, in addition to the subsequent creation of general categories ^(^ 15 ^,^ 19 ^)^ . These general categories emerged inductively were used to compose the variables of interest in the extraction instrument developed by the authors. 

 Subsequently, the content listed among the general categories was grouped according to identical, similar and complementary ideas, a refinement that contributed to updating the categories. The deductive emergence of the new grouped categories enabled a systematic organization of results, simplifying the understanding of information and facilitating the identification of trends, patterns and relevant insights related to the research question in focus ^(^ 15 ^,^ 19 ^)^ . 

 The search strategy and results of the selection process were presented in a PRISMA-ScR flowchart ^(^ 14 ^)^ . The extracted data was presented in a descriptive and tabular format, observing the JBI guidelines ^(^ 15 ^)^ . Additionally, synthesis images and a worldwide choropleth map were generated, representing the percentage of articles published by country, with the aim of presenting the results in a visual and graphical way. 

It should be noted that ChatGPT was not used in the textual preparation of this article, nor as a co-author responsible for the content, but only as an auxiliary tool in the spelling and grammar review of part of the sections of the manuscript.

## Results

**Figure 1:**
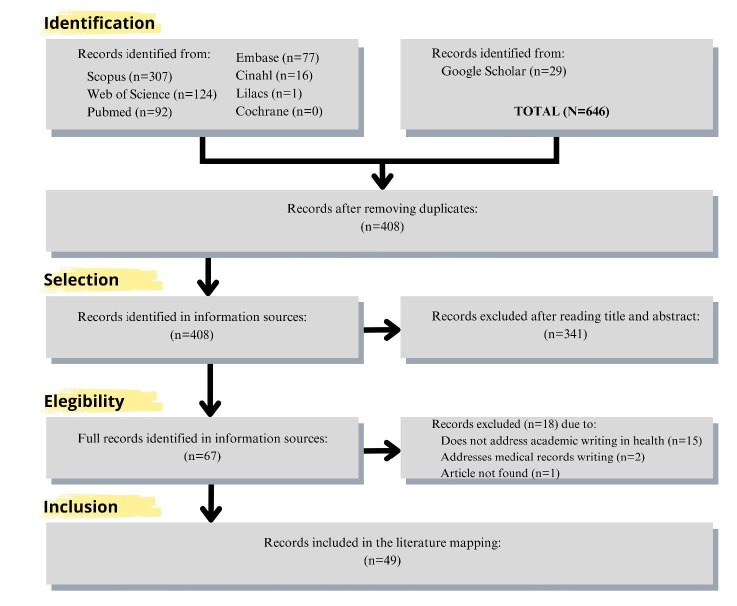
- Flowchart of the study selection process according to the Preferred Reporting Items for Systematic reviews and Meta-Analyses extension for Scoping Reviews (PRISMA-ScR). Ribeirão Preto, SP, Brazil, 2023

 Initially, 646 studies were found in databases and gray literature. After removing duplicates, 408 potentially eligible publications remained. The analysis of titles and abstracts led to the exclusion of 341 documents, resulting in the selection of 67 articles for full reading. After a rigorous selection process, 49 articles composed the final sample, as represented in Figure 1 . 

### Studies characteristics

The present article focuses on the wide range of contexts in which ChatGPT has been employed, as evidenced by analysis of the 49 articles included in this review. The authors explored several application areas, ranging from issues related to scientific integrity to generating scientific summaries and conducting systematic reviews.

**Figure 2:**
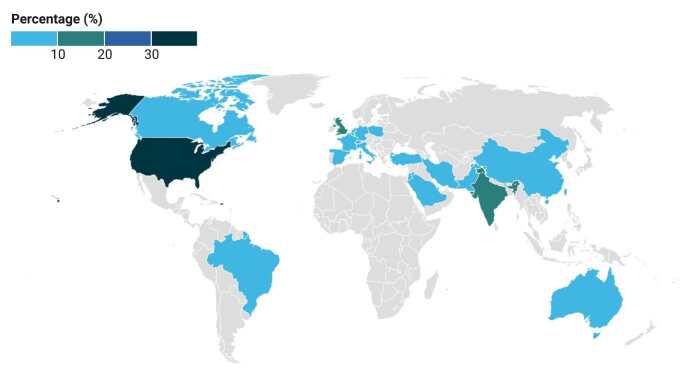
- Frequency distribution of articles published by countries. Ribeirão Preto, SP, Brazil, 2023

 The thematic map presented in Figure 2 offers a proportional choropleth representation of the distribution of the articles, considering the authors’ countries of origin, with emphasis on the United States of America (USA), India and the United Kingdom. 

Absolute frequency (N) and relative frequency (%): United States of America (23; 32.39%); India and the United Kingdom (8; 11.27%); Singapore and the Netherlands (3; 4.23%); China, Germany, Canada, Spain, Pakistan, Australia and Qatar (2; 2.82%); Saudi Arabia, Turkey, Brazil, France, Jordan, Belgium, Switzerland, Taiwan, Scotland, Iran, Poland and Italy (1; 1.41%)

 One of the articles was published in German, while the other 48 works were available in English, as shown in Figure 3 . In addition to this information, other details are available, such as types of sources, article titles and years of publication of the 49 articles included in this review. 

**Figure 3:** - Characterization of articles included in the scoping review. Ribeirão Preto, SP, Brazil, 2023

**Type of source**	**Article title**	**Language**	**Year**
Opinion article ^(^ 20 ^)^	ChatGPT ^*^ and the future of medical writing	English	2023
Opinion article ^(^ 21 ^)^	ChatGPT ^*^ : Disruptive Educational Technology	English	2023
Opinion article ^(^ 22 ^)^	ChatGPT ^*^ and other artificial intelligence chatbots and biomedical writing	English	2023
Opinion article ^(^ 23 ^)^	Using ChatGPT ^*^ in the Medical Field: A Narrative	English	2023
Opinion article ^(^ 24 ^)^	ChatGPT ^*^ and publication ethics	English	2023
Original article ^(^ 25 ^)^	Artificial intelligence in scientific writing: a friend or a foe?	English	2023
Original article ^(^ 26 ^)^	Generative artificial intelligence: Can ChatGPT ^*^ write a quality abstract?	English	2023
Original article ^(^ 27 ^)^	From human writing to artificial intelligence generated text: examining the prospects and potential threats of ChatGPT ^*^ in academic writing	English	2023
Original article ^(^ 28 ^)^	Implications of large language models such as ChatGPT ^*^ for dental medicine	English	2023
Original article ^(^ 29 ^)^	ChatGPT ^*^ for Future Medical and Dental Research	English	2023
Original article ^(^ 30 ^)^	Comparing scientific abstracts generated by ChatGPT ^*^ to real abstracts with detectors and blinded human reviewers	English	2023
Original article ^(^ 31 ^)^	Heat and Moisture Exchanger Occlusion Leading to Sudden Increased Airway Pressure: A Case Report Using ChatGPT ^*^ as a Personal Writing Assistant	English	2023
Original article ^(^ 32 ^)^	Extraventricular Neurocytoma of the Posterior Fossa: A Case Report Written by ChatGPT ^*^	English	2023
Original article ^(^ 33 ^)^	The role of ChatGPT ^*^ in scientific communication: writing better scientific review articles	English	2023
Original article ^(^ 34 ^)^	Pushing the Boundaries of Scientific Research with the use of Artificial Intelligence tools: Navigating Risks and Unleashing Possibilities	English	2023
Original article ^(^ 35 ^)^	ChatGPT ^*^ and a new academic reality: Artificial Intelligence-written research papers and the ethics of the large language models in scholarly publishing	English	2023
Original article ^(^ 7 ^)^	Can ChatGPT ^*^ draft a research article? An example of population-level vaccine effectiveness analysis	English	2023
Original article ^(^ 36 ^)^	Personality Changes and Staring Spells in a 12-Year-Old Child: A Case Report Incorporating ChatGPT ^*^ , a Natural Language Processing Tool Driven by Artificial Intelligence (AI ^†^ )	English	2023
Original article ^(^ 11 ^)^	ChatGPT ^*^ Utility in Healthcare Education, Research, and Practice: Systematic Review on the Promising Perspectives and Valid Concerns	English	2023
Original article ^(^ 37 ^)^	Can artificial intelligence help for scientific writing?	English	2023
Original article ^(^ 38 ^)^	Early applications of ChatGPT ^*^ in medical practice, education and research	English	2023
Original article ^(^ 39 ^)^	Artificial intelligence: How will ChatGPT ^*^ and other AI ^†^ applications change our everyday medical practice?	German	2023
Original article ^(^ 40 ^)^	Comparing human and artificial intelligence in writing for health journals: an exploratory study	English	2023
Original article ^(^ 41 ^)^	ChatGPT ^*^ : Is this version good for healthcare and research?	English	2023
Original article ^(^ 42 ^)^	ChatGPT ^*^ for research and publication: Opportunities and challenges	English	2023
Original article ^(^ 43 ^)^	Chatbots, ChatGPT ^*^ , and Scholarly Manuscripts: WAME ^‡^ Recommendations on ChatGPT ^*^ and Chatbots in Relation to Scholarly Publications	English	2023
Letter to the editor ^(^ 44 ^)^	Chatbots in Medical Research: Advantages and Limitations of AI ^†^ -Enabled Writing With a Focus on ChatGPT ^*^ as an Author	English	2022
Comment ^(^ 45 ^)^	ChatGPT ^*^ in Scientific Writing: A Cautionary Tale	English	2023
Brief communication ^(^ 46 ^)^	ChatGPT ^*^ in academic publishing: An ally or an adversary?	English	2023
Brief communication ^(^ 47 ^)^	Artificial Intelligence and new language models in Ophthalmology: Complications of the use of silicone oil in vitreoretinal surgery	English	2023
Editorial ^(^ 48 ^)^	The rise of AI ^†^ co-authors: navigating the future of scientific writing with ChatGPT ^*^	English	2023
Editorial ^(^ 49 ^)^	Chatbots and ChatGPT ^*^ - Ethical considerations in scientific publications	English	2023
Editorial ^(^ 50 ^)^	Artificial Hallucinations in ChatGPT ^*^ : Implications in Scientific Writing	English	2023
Editorial ^(^ 51 ^)^	ChatGPT ^*^ and other artificial intelligence applications speed up scientific writing	English	2023
Editorial ^(^ 6 ^)^	Artificial intelligence bot ChatGPT ^*^ in medical research: the potential game changer as a double-edged sword	English	2023
Editorial ^(^ 52 ^)^	Technological Impacts on the Sphere of Professional Journals	English	2023
Editorial ^(^ 53 ^)^	Nonhuman “Authors” and Implications for the Integrity of Scientific Publication and Medical Knowledge	English	2023
Editorial ^(^ 54 ^)^	Authorship and ChatGPT ^*^	English	2023
Editorial ^(^ 55 ^)^	ChatGPT ^*^ and scientific publications: friend or foe?	English	2023
Editorial ^(^ 56 ^)^	Pros and Cons of using ChatGPT ^*^ in scientific writing: as it identifies for itself	English	2023
Editorial ^(^ 57 ^)^	Open artificial intelligence platforms in nursing education: Tools for academic progress or abuse?	English	2023
Editorial ^(^ 58 ^)^	ChatGPT ^*^ : the new panacea of the academic world	English	2023
Editorial ^(^ 59 ^)^	Elevating scientific writing with ChatGPT ^*^ : A guide for reviewers, editors…and authors	English	2023
Editorial ^(^ 44 ^)^	Chatbots in Medical Research: Advantages and Limitations of Artificial Intelligence–Enabled Writing With a Focus on ChatGPT ^*^ as an Author	English	2022
Editorial ^(^ 60 ^)^	ChatGPT ^*^ as an author of academic papers is wrong and highlights the concepts of accountability and contributorship	English	2023
Editorial ^(^ 61 ^)^	The rise of artificial intelligence: addressing the impact of large language models such as ChatGPT ^*^ on scientific publications	English	2023
Editorial ^(^ 62 )	NLP § systems such as ChatGPT ^*^ cannot be listed as an author because these cannot fulfill widely adopted authorship criteria	English	2023
Editorial ^(^ 63 )	A Ghostwriter for the Masses: ChatGPT * and the Future of Writing	English	2023
Preprint ^(^ 64 )	Integrating chatbots (ChatGPT * ) in the process of manuscript writing and proposing a roadmap for their future adoption	English	2023

* ChatGPT = Chat Generative Pre-trained Transformer;

† AI = Artificial Intelligence;

‡ WAME = World Association of Medical Editors;

§ NLP = Natural Language Processing

 More information about the publications analyzed and that composed the corpus of this study can be found in the Supplementary Material (available at: https://doi.org/10.48331/scielodata.BMQMKD ). 

### Using ChatGPT in academic and scientific writing

 According to the studies analyzed, ChatGPT proved to be versatile in different contexts. In the context of scientific integrity, ChatGPT demonstrated its ability to answer questions, highlighting its applicability in obtaining answers and information ^(^ 30 ^,^ 34 ^,^ 53 ^)^ . In the area of the pathogenesis of medical conditions, the model was evaluated for its ability to describe pathological processes in detail, contributing to a comprehensive understanding of these phenomena ^(^ 50 ^)^ . 

 Furthermore, its ability to generate simulated studies offered support in making methodological decisions and in the development of scientific manuscripts ^(^ 7 ^)^ . The generation of scientific summaries through ChatGPT has also demonstrated its ability to create summaries that meet the standards required by specific journals, using selected titles and journals as a basis ^(^ 26 ^,^ 30 ^,^ 38 ^,^ 47 ^)^ . 

 With regard to writing academic content, ChatGPT demonstrated its ability to contribute to the formulation of parts of articles, promoting significant improvements in clarity and textual cohesion ^(^ 28 ^-^ 29 ^,^ 38 ^,^ 40 ^)^ . Its ability to distinguish between machine-generated and human-generated text has proven valuable in evaluating AI texts and, consequently, in composing editorials, covering titles, introductions and supporting references ^(^ 28 ^)^ . 

 ChatGPT also facilitated the optimization of the writing of scientific articles itself, assisting in the creation of drafts, literature review and improvement of the language used ^(^ 7 ^,^ 11 ^,^ 25 ^-^ 26 ^,^ 29 ^-^ 30 ^,^ 33 ^,^ 40 ^-^ 41 ^)^ . Its influence has also been observed in systematic reviews, where its interaction with the tool has been examined ^(^ 11 ^)^ . 

 Regarding emerging themes, the frequency of the terms “use”, “writing”, “scientific writing”, “academic authorship”, “generation” and “impact” stands out. The topics covered range from scientific integrity and the evaluation of writing generation ^(^ 6 ^)^ , to specific medical cases, such as acute dacryocystitis ^(^ 49 ^)^ , intracranial neoplasia ^(^ 36 ^)^ , endometrial receptivity in *in vitro* fertilization ^(^ 25 ^)^ and plantar fasciitis in children ^(^ 26 ^)^ . 

 The exploration covers the use of ChatGPT in the generation, evaluation and editing of scientific texts ^(^ 23 ^,^ 30 ^-^ 31 ^,^ 33 ^,^ 43 ^,^ 58 ^,^ 65 ^-^ 66 ^)^ , as well as its application in journals, academic and medical writing ^(^ 32 ^,^ 38 ^,^ 50 ^)^ , production of abstracts ^(^ 30 ^)^ and health education ^(^ 11 ^,^ 38 ^)^ . The authors also reflect on the potential impact of ChatGPT on writing, considering its advantages and limitations, and speculate on the future of this tool in the development of scientific content ^(^ 11 ^,^ 24 ^,^ 34 ^-^ 35 ^,^ 37 ^,^ 55 ^-^ 56 ^,^ 59 ^,^ 63 ^)^ . Furthermore, the researchers undertook an analysis to determine whether this AI in question should be recognized and considered as a legitimate author in academic productions ^(^ 22 ^,^ 35 ^)^ . 

### Benefits, risks, concerns and limitations

**Figure 4:**
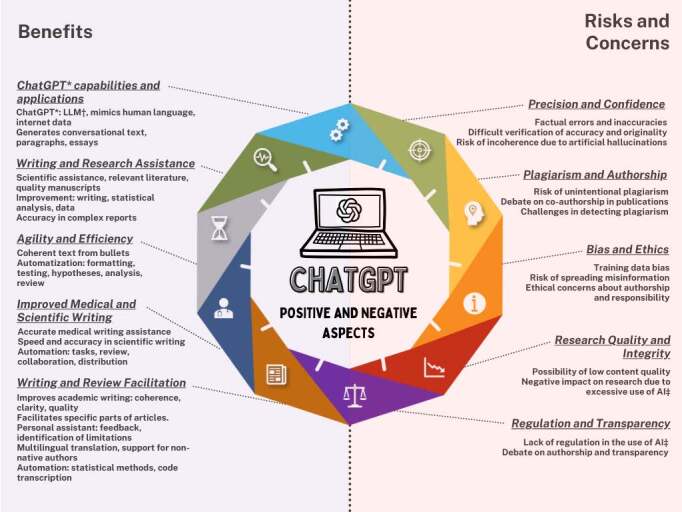
- Benefits, risks and concerns regarding the use of ChatGPT in academic writing. Ribeirão Preto, SP, Brazil, 2023

 In examining the set of articles analyzed, the authors not only portrayed a diversity of ChatGPT applications, but also highlighted their benefits, risks and underlying concerns. The summary image presented in Figure 4 helps to identify the positive and negative aspects described. 

*ChatGPT = Chat Generative Pre-trained Transformer; ^†^ LLM = Large Language Model; ^‡^ AI = Artificial Intelligence

**Figure 5:**
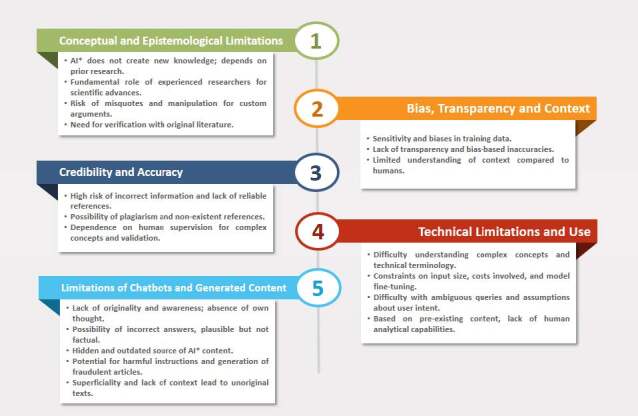
- Limitations associated with using ChatGPT in academic writing. Ribeirão Preto, SP, Brazil, 2023

 In addition to the risks and benefits associated with using ChatGPT in academic writing, the mapped literature also highlighted the limits and restrictions of the tool today. Figure 5 illustrates the limitations of using ChatGPT in academic writing. 

*AI = Artificial Intelligence

### Future perspectives

Finally, this review identified several perspectives related to the potential use of ChatGPT in academic writing. The analyzes revealed perspectives on several facets of this integration process, presented below:

*Structure and Responsibility* : The need to establish barriers and structures for the use of AI is highlighted, as well as assigning responsibility for authorship by chatbots. It is suggested that measures be adopted to fully exploit the potential of AI ^(^ 54 ^,^ 60 ^,^ 62 ^)^ . 

*Evolution of Chatbots and Positive Impact* : The rapid evolution of chatbots is observed, with the potential to overcome current limitations. ChatGPT is recognized for accelerating innovation, optimizing academic training, improving writing and analysis skills, and bringing benefits to healthcare and research ^(^ 34 ^,^ 50 ^,^ 54 ^)^ . 

*Modifications and Ethical Challenges* : It is proposed to modify manuscript evaluation policies and practices, including the adoption of AI output detectors. Ethical concerns and the need to balance positive and negative impacts on academic writing are emphasized ^(^ 11 ^,^ 24 ^,^ 29 ^-^ 30 ^,^ 35 ^,^ 47 ^,^ 49 ^)^ . 

*Regulation and Technical Improvements* : The importance of clear ethical guidelines and regulations for the use of AI is highlighted. The search for robust plagiarism detection tools and technical improvements is evidenced ^(^ 11 ^,^ 21 ^,^ 23 ^,^ 30 ^,^ 33 ^,^ 38 ^-^ 39 ^,^ 62 ^)^ . 

*Adaptation and Future of AI* : There is a growing trend towards integrating AI, including ChatGPT, into academic writing, highlighting the need for rapid adaptation by researchers and editors ^(^ 20 ^,^ 29 ^,^ 32 ^,^ 34 ^,^ 48 ^,^ 63 ^-^ 64 ^,^ 66 ^)^ . 

*Automation and Enhancement:* The potential for automating repetitive tasks and improving language through AI is highlighted, enabling statistical reviews and comprehensive research in the literature ^(^ 23 ^)^ . 

*Collaboration and Ethics* : The need for codes of ethics and guidelines for the responsible use of AI is emphasized, highlighting the importance of balancing automation and human supervision ^(^ 11 ^,^ 30 ^,^ 34 ^)^ . 

*Impact and Gradual Integration* : The revolutionary potential of ChatGPT in scientific writing is recognized, with a trend towards gradual integration into medicine and academic research, emphasizing responsible use and development ^(^ 28 ^,^ 30 ^,^ 33 ^-^ 35 ^,^ 50 ^,^ 52 ^,^ 61 ^)^ . 

*Disruptive Changes and Improvements* : Possible disruptive changes in science due to AI are observed, requiring adjustments in editorial policies and continuous improvements in ChatGPT, especially in medicine ^(^ 21 ^,^ 38 ^)^ . 

*Specific Development and Impact in the Health Area* : It is suggested that specific AI tools be developed for scientific needs, with attention to the potential impact on healthcare and collaboration between stakeholders ^(^ 11 ^,^ 28 ^,^ 30 ^,^ 33 ^-^ 35 ^,^ 40 ^,^ 50 ^,^ 52 ^,^ 61 ^)^ . 

These findings signal a future panorama regarding the needs, evolution, impacts and challenges of using ChatGPT in the academic context.

## Discussion

 The dominance of the US and UK in research on the use of ChatGPT in academic writing in health is due to their leadership in research, innovation and academic resources. These countries are highlighted in the Global Innovation Index 2022, leading the global innovation ranking. They are also home to renowned research and technology institutions, which encourages the exploration of technologies like ChatGPT, facilitating interdisciplinary collaborations. India, an emerging economy, is gaining prominence on the global innovation scene, and is among the 40 best-rated nations in the Global Innovation Index ^(^ 67 ^)^ . 

 The predominance of the English language in scientific studies is intrinsically linked to the success of researchers, whose trajectory depends on the production of scientific articles and the impact of the journals they publish. Given that most of the most prestigious journals are published in English, achieving research success is closely associated with publishing in that language ^(^ 68 ^)^ . Furthermore, it is worth mentioning that ChatGPT is English-based. 

 Regarding the contexts and topics covered, it was evident that the majority of studies have as their main focus the exploration of scientific writing through ChatGPT ^(^ 23 ^,^ 30 ^-^ 31 ^,^ 33 ^,^ 43 ^,^ 45 ^,^ 58 ^,^ 65 ^-^ 66 ^)^ . This reflects the growing quest to understand how this technology can impact and improve the production of scientific content, paving the way for discussions about its effectiveness, applicability and transformative potential in the academic field. 

 Regarding the positive aspects listed in studies concerning the use of ChatGPT, it is highlighted that ChatGPT offers benefits in academic and scientific writing, due to its speed, refined language and notable contribution ^(^ 11 ^,^ 26 ^,^ 28 ^,^ 36 ^)^ . This tool assists scientists in writing articles, speeds up the creation of documents with cohesive text generated from markers, improves medical and scientific writing ^(^ 25 ^,^ 46 ^)^ , facilitates the writing of specific sections of articles ^(^ 25 ^,^ 69 ^)^ , speeds up research and analysis, and promotes equity by assisting non-native English-speaking authors ^(^ 45 ^,^ 51 ^)^ . It is believed that ChatGPT can make the research and publishing process more efficient, being useful for researchers, journal editors and reviewers ^(^ 42 ^)^ . 

Therefore, ChatGPT functions as a multifaceted tool, generating high-quality ideas and essays, improving writing coherence and supporting scientific and medical writing. It also automates repetitive tasks in manuscript preparation. Its features include emulation of linguistic patterns from a vast database, fast and sophisticated responses, and support for hypothesis creation and data analysis.

 ChatGPT is relevant for improving writing, reducing redundancies, suggesting synonyms to enrich vocabulary and paraphrasing to modify the style of the text. Furthermore, it effectively handles large volumes of data, contributing to automation and innovation ^(^ 6 ^,^ 51 ^)^ . It also assists in restructuring manuscripts, offers feedback and speeds up the writing process, being applicable in clinical reports, improving the quality of radiology reports and complex abstracts ^(^ 6 ^)^ . 

Thus, in the set of articles analyzed, ChatGPT emerges as a valuable tool for researchers and scientists by improving writing, speeding up tasks, enhancing quality and offering support in various areas, boosting the efficiency and quality of academic and scientific production.

 Assessing the limitations of ChatGPT in academic writing reveals significant challenges. AI cannot generate original knowledge, requiring prior research and collaboration from experienced researchers. Concerns include the possibility of incorrect citations and manipulation of chatbot output for personalized argumentation, emphasizing the importance of critical analysis of generated text and verification with original literature. There are also limitations in understanding specialized terminology and a lack of a clear methodology for selecting and citing sources ^(^ 6 ^,^ 45 ^,^ 63 ^,^ 70 ^)^ . 

 It is essential to note that references provided by ChatGPT are currently unreliable and require detailed review. In situations where ChatGPT has no response, it may generate fictitious output called “hallucination”, providing false information such as authors, titles, and article DOIs. To obtain reliable answers, it is necessary to train the language model on specific knowledge domains, a complex and expensive process. Stanford researchers and the company MosaicML are collaborating on the development of a model called PubMed GPT, but balancing the model’s complexity, costs, and need for specialized architecture is challenging ^(^ 25 ^,^ 49 ^,^ 63 ^,^ 71 ^)^ . 

 An additional set of limitations is related to the inherent characteristics of chatbots. These systems have limitations, including a lack of originality and responses that are not always truthful, due to outdated or non-transparent AI sources. This raises concerns about the reliability of the generated content and, by repeating a question, different answers may be generated. The risk of disseminating incorrect information makes the use of AI in academic production complex, highlighting challenges in the accuracy and credibility of the content generated ^(^ 50 ^,^ 72 ^)^ . Figure 5 presents a summary of the limitations associated with the use of ChatGPT in academic writing, and evidences the need for careful analysis of what is produced by this technology, in order to mitigate biases and inaccuracies. 

 Several academic actors, including editors of journals, periodicals and scientific institutions, such as WAME (World Association of Medical Editors), COPE (Committee on Publication Ethics) and the JAMA network, have highlighted the role of AI in scientific publications. COPE’s official position is enlightening in this context, as it emphasizes that AI tools cannot satisfy authorship criteria, as they cannot take responsibility for submitted content. As non-legal entities, they cannot assert the presence or absence of conflicts of interest, nor manage issues related to copyright and licenses. In appropriate situations, the chatbot can be recognized, but it is not allowed to assign any author status to it ^(^ 49 ^,^ 73 ^)^ . 

 It is crucial to understand that in the context of scientific publishing, an author is not just someone who writes a document, but a fundamental participant in an academic enterprise. According to the International Committee of Medical Journal Editors (ICMJE), chatbots do not meet authorship criteria, especially with regard to the ability to give “final approval of the version to be published” and assume responsibility for all aspects of the work to ensure accuracy and integrity. Authorship guidelines in scientific literature are strict, and chatbots do not meet these criteria, as AI cannot consent to being an author or take responsibility for its contributions ^(^ 62 ^)^ . 

 Therefore, it is important to highlight WAME ^(^ 43 ^)^ recommendations on “chatbots and generative artificial intelligence in relation to academic publishing”: 1) Chatbots cannot be authors, as these tools cannot be held responsible for any statement or any ethical violation; 2) Authors must be transparent when chatbots are used and provide information about how they were used. The extent and type of use of chatbots in journal publications should be indicated. This is consistent with the ICMJE recommendation to acknowledge written assistance and provide in the methods detailed information about how the study was conducted and the results generated. 

 In the context of using AI, it is crucial to establish specific guidelines to ensure the integrity and transparency of the results presented in scientific articles. To this end, it is essential that authors provide detailed information about the prompts used when employing AI in tasks such as analyzing data, creating tables, figures, or writing code. This information should be clearly described in the abstract and methods section of the article, including date, time, AI tool used and its corresponding version, in order to enable scientific scrutiny and replication of results ^(^ 11 ^,^ 30 ^,^ 34 ^)^ . 

 Furthermore, authors have the responsibility to ensure the accuracy of the content generated by chatbots in their articles, guaranteeing the absence of plagiarism and correctly attributing all sources, including the originals of the material generated by the chatbot. Editors and reviewers play a fundamental role when evaluating manuscripts that use chatbots, and must clearly communicate with each other and with the authors whether they used this technology in analyzing and reviewing articles. If they have used chatbots in their communications, it is important that they explain the context and purpose of this use ^(^ 11 ^,^ 30 ^,^ 34 ^)^ . 

 Finally, publishers must have effective tools to detect content generated or altered by AI, which must be available in an accessible way, regardless of financial resources. This measure is essential to preserve the integrity of information related to healthcare and mitigate possible risks to public health ^(^ 11 ^,^ 30 ^,^ 34 ^)^ . 

 Furthermore, when using ChatGPT in academic writing, it is crucial to deal with ethical issues such as harmful instructions and the production of fraudulent content, as well as the manipulation or fabrication of images ^(^ 49 ^)^ . In this sense, human supervision is necessary, especially in fields with complex concepts and scientific subtleties, ensuring the accuracy and legitimacy of information and statements, and preserving the integrity of research and academic production. Discussions about potential biases in training data and lack of transparency highlight the need for caution and rigorous analysis when applying ChatGPT to scientific writing ^(^ 25 ^)^ . 

 These challenges can affect the accuracy and reliability of the information generated, emphasizing the need for caution when incorporating ChatGPT into academic writing ^(^ 47 ^)^ . Besides, the superficiality and lack of originality of the content produced by ChatGPT is evident, often due to the lack of context and AI expertise. Therefore, the inability to generate up-to-date content and innovative ideas also presents as a significant limitation, as well as the difficulty in understanding highly specialized areas such as human anatomy and medical information ^(^ 11 ^,^ 30 ^)^ . 

 The set of future perspectives covered in the articles explores the various aspects of integrating ChatGPT into academic writing. The need to establish barriers and structures for the appropriate use of AI emerges, while emphasizing the importance of assigning responsibility for authorship to chatbots and adopting measures to fully harness their potential ^(^ 49 ^)^ . The rapid evolution of chatbots indicates the potential to overcome current limitations, highlighting ChatGPT as a tool that accelerates innovations, optimizes academic training and improves writing and analysis skills ^(^ 74 ^)^ . 

 The perspectives also consider the need to reformulate evaluation policies, which include AI-generated result detectors, with a sharp focus on ethical issues and the search for a balance between benefits and challenges. This highlights the importance of clear ethical guidelines and regulations for AI, along with the continuous search for technical improvements and plagiarism detection tools ^(^ 9 ^,^ 75 ^)^ . Tools like DetectGPT and Orginality.ai are coming to market to try to detect AI-written content ^(^ 49 ^,^ 63 ^)^ , in addition to GPTZero ^(^ 76 ^)^ . 

 The use of ChatGPT in writing scientific essays has proven to be effective, however, it is important to recognize that the data generated by this tool consists of a combination of true and completely fictitious information. This duality raises significant concerns about the integrity and accuracy of using large language models like ChatGPT in academic writing ^(^ 25 ^,^ 33 ^,^ 48 ^)^ . Given this scenario, a review of the policies and practices for evaluating scientific manuscripts for journals and conferences in the health sector is suggested, in order to maintain rigorous scientific standards. 

 The discussion surrounding the use of extensive language models in scientific writing also raises ethical and acceptability questions. Additionally, there are concerns about the possibility of creating fake medical experts through AI, which could pose a risk due to the lack of real experience and the generation of opinions from supposed experts through ChatGPT (or similar AI). These considerations highlight the importance of an ongoing debate on the responsible use of AI in the production of scientific and medical content ^(^ 50 ^)^ . 

 The trend towards integrating AI, more precisely ChatGPT, into academic writing is highlighted, showing the need for researchers to adapt and emphasizing the importance of human collaboration and supervision. Additionally, the relevance of AI in the healthcare context is emphasized, with suggestions for developing specific tools to meet scientific needs, along with careful attention to its impact on healthcare and research. This trend is corroborated by several studies ^(^ 11 ^,^ 20 ^,^ 27 ^-^ 28 ^,^ 37 ^,^ 40 ^,^ 45 ^,^ 52 ^,^ 56 ^,^ 66 ^)^ . 

Considering the important methodological limitations that must be taken into account, this study requires a critical analysis. Firstly, it is necessary to recognize that the scoping analysis carried out may not have covered all potential applications and contexts of AI in healthcare, due to the constant technological evolution in this field. Besides, the predominance of studies with a low level of evidence, based on reflections, experience reports and editorials, raises concerns about the robustness of the conclusions. Therefore, there is an urgent need to conduct more robust, evidence-based research to assess the impact of AI on healthcare and provide solid guidance for clinical decisions.

Moreover, other limitations of the study refer to the sources of information and databases, since the scoping review is comprehensive. In the sources identification step, comprising libraries and repositories, the detailed description of certain bases, which could be present in one or more of these information sources, was not undertaken in this study. However, this review stands out for the methodological rigor established by JBI, which reinforces the credibility of the findings presented.

By exploring the potential use of ChatGPT in academic writing, this research provides a deeper understanding of how AI can be applied to improve the production of academic content, ranging from scientific articles to healthcare documents. This can result in greater efficiency in creating academic materials, while also highlighting the importance of addressing ethical and quality issues when integrating AI in this context. Thus, this study significantly contributes to the understanding of the opportunities and challenges related to AI in the production of scientific knowledge in health and nursing.

## Conclusion

The present study focused on mapping the potential use of ChatGPT in academic writing in health, addressing both its benefits and associated ethical concerns. In this context, this research contributes significantly to knowledge, as it highlights the disruptive nature of chatbots and the importance of effective integration of these tools in scientific publishing, while recognizing the limitations and risks involved in using AI in writing.

In short, we can conclude that ChatGPT has transformative potential in research and academic writing in the health area. However, its implementation must be accompanied by rigorous human supervision, solid regulations, and transparent guidelines to ensure its responsible integration into the scientific community. As healthcare and nursing are already being influenced by AI, it is essential to anchor this trajectory in responsibility and ethical use, especially when employing ChatGPT in academic writing in health.
